# Isolation of HIV-1-Neutralizing Mucosal Monoclonal Antibodies from Human Colostrum

**DOI:** 10.1371/journal.pone.0037648

**Published:** 2012-05-18

**Authors:** James Friedman, S. Munir Alam, Xiaoying Shen, Shi-Mao Xia, Shelley Stewart, Kara Anasti, Justin Pollara, Genevieve G. Fouda, Guang Yang, Garnett Kelsoe, Guido Ferrari, Georgia D. Tomaras, Barton F. Haynes, Hua-Xin Liao, M. Anthony Moody, Sallie R. Permar

**Affiliations:** 1 Human Vaccine Institute, Duke University Medical Center, Durham, North Carolina, United States; 2 Department of Immunology, Duke University Medical Center, Durham, North Carolina, United States; Mayo Clinic, United States of America

## Abstract

**Background:**

Generation of potent anti-HIV antibody responses in mucosal compartments is a potential requirement of a transmission-blocking HIV vaccine. HIV-specific, functional antibody responses are present in breast milk, and these mucosal antibody responses may play a role in protection of the majority of HIV-exposed, breastfeeding infants. Therefore, characterization of HIV-specific antibodies produced by B cells in milk could guide the development of vaccines that elicit protective mucosal antibody responses.

**Methods:**

We isolated B cells from colostrum of an HIV-infected lactating woman with a detectable neutralization response in milk and recombinantly produced and characterized the resulting HIV-1 Envelope (Env)-specific monoclonal antibodies (mAbs).

**Results:**

The identified HIV-1 Env-specific colostrum mAbs, CH07 and CH08, represent two of the first mucosally-derived anti-HIV antibodies yet to be reported. Colostrum mAb CH07 is a highly-autoreactive, weakly-neutralizing gp140-specific mAb that binds to linear epitopes in the gp120 C5 region and gp41 fusion domain. In contrast, colostrum mAb CH08 is a nonpolyreactive CD4-inducible (CD4i) gp120-specific mAb with moderate breadth of neutralization.

**Conclusions:**

These novel HIV-neutralizing mAbs isolated from a mucosal compartment provide insight into the ability of mucosal B cell populations to produce functional anti-HIV antibodies that may contribute to protection against virus acquisition at mucosal surfaces.

## Introduction

Anti-HIV-1 neutralizing antibodies are induced late following primary infection, rendering these antibodies ineffective in controlling viral replication [1]. However, passive infusion of broadly-neutralizing antibodies isolated from HIV-1-infected individuals can protect against virus acquisition [2], [3], [4], [5], [6]. Unfortunately, HIV-1 vaccine strategies have failed to induce broadly-neutralizing antibody responses. Thus, it is important to characterize naturally-elicited HIV-1-neutralizing antibodies, identifying potent anti-HIV-1 antibodies that may be readily-induced by vaccination.

Some studies suggest that neutralizing antibodies are present in mucosal sites of HIV-1-exposed in individuals who remain seronegative [7], [8], making mucosal compartments a potential source of HIV-1-neutralizing antibodies. However, mAb isolation from mucosal sites has not previously been established, partially due to the difficulty obtaining adequate mucosal specimens. Breast milk is an ideal source for isolation of mucosal antibodies, as it is easily collected and rich in immunoglobulin-secreting B cells [9] that originate in the gastrointestinal-associated lymphoid tissue [10], [11], [12]. The specificity and function of these mucosal antibodies may be distinct from those in plasma [9], [13], [14]. Moreover, functional HIV-1 Env-specific antibodies are elicited in milk during chronic infection [13] and following systemic vaccination [15]. Thus, characterization of monoclonal antibodies produced by milk B cells will reveal unique insight into the induction of anti-HIV-1 mucosal B cell responses. Therefore, we sought to identify and characterize functional anti-HIV-1 Env-specific antibodies produced by B cells isolated from colostrum of an HIV-1-infected, lactating mother with a strong milk neutralization response. In this work, we demonstrate that mucosal B cells are capable of producing HIV-1-neutralizing antibodies, an important goal of effective HIV-1 vaccination.

## Materials and Methods

### Ethics statement

This study (CHAVI009) was approved by the Division of Acquired Immunodeficiency Syndrome, National Institute of Allergy and Infectious Diseases, National Institute of Health (DAIDS-ES ID 10491); the College of Medicine Research and Ethics Committee in Malawi (P.06/06/440); and institutional review boards at each participating institution, including University of North Carolina (07–0831), Duke University (Pro00003582), and Beth Israel Deaconess Medical Center (2006_P_000199). Written consent was obtained from each subject.

### Subject and colostrum processing

Colostrum was collected from a chronically HIV-1-infected, lactating African woman at 3 days postpartum. A single dose of nevirapine was given to the mother and infant at delivery. Colostrum cells were isolated [16] and cryopreserved within 6 hours of sample collection.

### B cell isolation and immunoglobulin (Ig) production and screening

Memory B cell isolation was performed as described [17], [18] with the following modifications. Thawed colostrum cells were stained with a viability marker (Aqua Vital Dye) and the following antibodies: CD27 pacific blue, anti-IgM FITC, anti-IgD PE, CD3 PE-Cy5, CD16 PE-Cy5, CD235a PE-Cy5, CD5 PE-Cy7, CD19 APC-Cy7 (BD Biosciences); CD38 APC-Alexa Fluor 700 (Beckman Coulter); CD14 PE-Cy5 (Invitrogen). Total B cells were gated as viable (Aqua Vital Dye−) CD3/CD14/CD16/CD235a−, and CD19+; memory B cells were further selected by gating on surface IgD− cells. Cell sorting was performed using a FACSAria2 (BD Biosciences) as single-cells into 96-well plates precharged with a RNA stabilization cocktail and subjected to immunoglobulin gene RNA amplification, as previously described [17], [19]. Ig isotype was determined by sequence homology and inferred rearrangement of variable, diversity, and joining (VDJ) regions was performed using SoDA [20]. Estimates of complementarity determining region 3 of the heavy chain (CDRH3) charge and hydropathy, using the grand average of hydropathy (GRAVY) method, were determined using available tools [21]. Overlapping PCR was used to construct full length IgG1 (for heavy chain) and kappa or lambda (for light chain) cassettes for expression of recombinant antibodies [17], [19]. Selected antibodies were cloned into pcDNA 3.3 (Invitrogen) and co-transfected into 293T cells using polyethylenimine [22] for large-scale production.

### HIV-1 Env binding

The specificity of transfected cell culture supernatants and purified recombinant mAbs were assessed by ELISA [17] and multiplex antibody binding assays using Luminex, as previously described [23]. HIV-1 Envs used for screening included: consensus gp140 constructs from subtypes A, B, C, CRF01_AE, and G; and group M consensus gp140_Con.S_ and gp120_Con.6_ [24]. Screening was also performed with the following specific HIV-1 isolate constructs: gp140_00MSA.A_, gp140_VRC.A_, gp41_MN.B_, gp120_A244.01.AE_, gp120_MN.B_, gp140_VRC.B_, and gp140_DRCBL.G_. Antibodies palivizumab (against the F protein of respiratory syncytial virus [25], MedImmune) and CH96 (against influenza hemagglutinin, mAb 1248 in [26]) served as negative controls to determine the median fluorescence intensity (MFI) cut off of binding specificity. HIV-1 Env binding by mAbs was assessed at a range of concentrations (0.1 to 50 µg/ml) to determine the effective concentration 50% (EC_50_) via 5-parameter logistic regression curves using the *drc* analysis package [27].

### Linear epitope binding

Peptide microarray was performed with modifications from a previously-reported protocol using a Tecan HS4000 Hybridization Workstation [23]. Peptide libraries (designed by Dr. Bette Korber) consisting of 15-mers overlapped by 12 amino acids were printed onto glass slides, covering the full length of consensus gp160 Env from clades A, B, C, D, CRF01_AE, and CRF02_AG. Signal intensity of each spot was defined as the median 645 nm foreground measurement after background subtraction; defined as the belt located 3× the diameter around each spot.

### Linear C5 and fusion domain binding assay

Antibody binding to the HIV-1 Env gp120 C5 region and gp41 fusion domain peptide variants identified in the linear epitope binding assay was confirmed via surface plasmon resonance (SPR). Hydrophilicity and net charge of each peptide used in this analysis were calculated with the online Innovagen Peptide Property Calculator (http://www.innovagen.se/custom-peptide-synthesis/peptide-property-calculator/peptide-property-calculator.asp). Binding measurements were determined on a BIAcore 4000 instrument. Flow cells were activated with a solution of N-hydroxysuccinimide/N-(3-dimethylaminopropyl)-N′-ethylcarbodiimide. Fusion domain peptides in 10 mM sodium acetate buffer, pH 5.5 were immobilized to approximately 200 response units (RU). SP62 peptide was immobilized in 10 mM sodium acetate buffer, pH 4.0 to approximately 100 RU as a reference surface to subtract nonspecific responses. After immobilization, ethanolamine-HCl was used to block any remaining active sites on the chip surface. MAb CH07 (100 µg/mL) was injected at a flow rate of 30 µL/min for 3 minutes. After 300 seconds of dissociation, glycine (pH 2.0) was injected to regenerate chip surfaces. Data analysis was performed using BIAcore 4000 evaluation Software v. 1.0.

### Conformational Env binding assay

A soluble CD4-induced (sCD4i) Env binding assay was performed by SPR using a BIACore 3000 as previously described [28]. The following ligands were immobilized on a CM5 sensor chip: biotinylated rat IgG (BD Biosciences), HIV-1 Env-specific mAbs A32 (gift of James Robinson, Tulane School of Medicine) and T8 (gift of Pat Earl, NIH), and sCD4 (NIH AIDS reagent program, James Bradac, NIH) [29]. Env proteins (gp140_Con.S_ or gp120_JRFL.B_) were run over the immobilized ligands, followed by mAb CH08 (100 µg/mL). Data analyses were completed with BIAevaluation 4.1 software.

### Competitive inhibition/antibody blocking assay

A modified D7324 capture assay [29], [30] was used to confirm the specificity of mAb CH08. HIV-1 Env (0.08 µg gp140_Con.S_) was captured onto a plate using 0.03 µg of gp120 carboxy-terminus binding mAb 3B3. Wells were incubated with 0.05 µg of sCD4, followed by CD4i gp120-binding antibodies, 17b and 21C (gifts from James Robinson), and 412-D (gift from Susan Zolla-Pazner, New York University School of Medicine), followed by biotinylated mAb CH08. CH08-binding was detected with the addition of streptavidin-horseradish peroxidase and substrate; and optical density (OD) measured at 450 nm. Percent CH08-binding was calculated by subtracting the blank OD, then dividing ODs of the wells containing blocking antibody by wells with no blocking antibody.

### Neutralization assays

HIV-1 Env pseudovirus neutralization was assessed as described [31] in TZM-bl cells. Data is presented as the inhibitor concentration (IC_50_) that resulted in a 50% reduction of relative light units with respect to virus-only wells. In assays with breast milk and plasma, HIV-1 Env pseudoviruses were produced with an RT-resistant backbone (1617RT/K103N) and SVA.MLV Env pseudovirus was used as a negative control.

### Sulfation site identification

Sulfation sites within the CDR3 regions of CH07 and CH08 were predicted using the online sulfation prediction programs Sulfosite and Expasy Proteomics Server (Swiss Institute of Bioinformatics). To confirm the presence of a sulfation site, 2 µg of 412-D, CH08, and CH07 were sized on a 4–12% Bis-Tris Gel under reducing conditions. Anti-sulfotyrosine antibody (clone Sulfo-1c-A2, Millipore) was diluted 1∶500 in casein blocker and incubated overnight with the gel. Alkaline phosphatase-conjugated goat anti-mouse IgG (SouthernBiotech) diluted 1∶50,000 was used for detection.

### Antibody Dependent Cellular Cytotoxicity (ADCC) and virus capture assays

ADCC activity was measured using a GranToxiLux (OncoImmunin, Inc.) protocol as described [13], [32]. CEM.NKR_CCR5_ cells (A1953.B HIV-1-infected, CM243.E HIV-1-Env coated, and non-coated/uninfected) were incubated in the presence of mAb and PBMCs; ADCC activity is reported as the background-subtracted percent of CEM.NKR_CCR5_ cells positive for Granzyme B. Virus capture was assessed using mAb-coated plates as described [33]. Env-pseudotyped viruses SF162.B, BG1168.B, and CAP45.C were captured in the presence or absence of sCD4. Following lysis, captured virus was quantitated by p24 ELISA (Abbot Laboratories).

### Hep-2 epithelial binding, autoantigen microchip analysis, and lipid binding

Indirect immunofluorescence binding of mAbs to HEp-2 cells (Inverness Medical Professional Diagnostics) was performed as described [34]. Layout and scaling of images was performed in Adobe Photoshop without manipulation. The reactivity of mAb CH07 to a panel of ∼9000 full-length human proteins purified under native conditions was performed with a protein array microchip (ProtoArray v5.0, Invitrogen) according to manufacturer instructions. After blocking, microarrays were blotted with 2 µg/mL of mAb CH07 or human myeloma IgG (Southern Biotechnology Associates) for 90 min. Protein-antibody interactions were detected using 1 µg/mL anti-human IgG conjugated with Alexa Fluor 647. The arrays were scanned at 635 nm with 5-µm resolution, using 100% power, and 600 gain (GenePix 4000B scanner, Molecular Devices). Manufacturer-provided lot-specific protein spot definitions were aligned. Fluorescence intensities were quantified using GenePix Pro 5.0 (Molecular Devices) after subtraction of background. The results of mAb CH07-binding were compared to human myeloma isotype controls to determine fold change of fluorescence intensity and fluorescence. The binding of CH07 and CH08 to lipid mixtures composed of phosphatidylcholine-cardiolipin (25∶74 molar ratio) or phosphatidylcholine-phosphatidylserine (25∶75 molar ratio) was measured via SPR, as described [35].

## Results

### Isolation of HIV-1 Env-specific mAbs by B cell sorting of colostrum cells

B cells were isolated from colostrum from an HIV-1-infected woman (viral load: 60,335 copies/mL, CD4 count: 452) with detectable low-level neutralizing activity (range of ID_50_: 12–21) in breast milk against a panel of 4 tier 2-like breast milk Env variant pseudoviruses [36] that was greater than two fold above the nonspecific neutralization activity of milk of uninfected mothers [13]. Remarkably, no neutralization was detected against these viruses by a concurrently-collected plasma sample at a dilution of 1∶20. This milk neutralization response is notable, as tier 2 neutralization is infrequently detected in milk and is typically at least two-fold lower than that in plasma [13]. The infant of this mother remained HIV-1 uninfected (determined by blood HIV-1 DNA PCR) after 6 months of breastfeeding.

**Figure 1:**
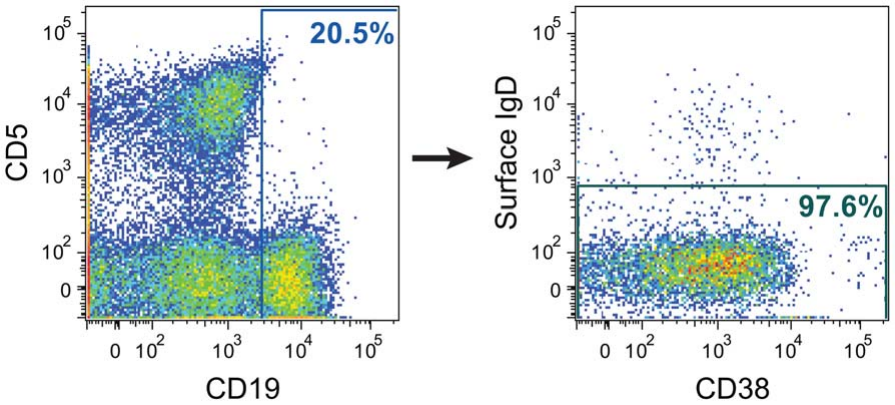
Memory B cell isolation from colostrum of an HIV-1-infected, lactating woman. Colostrum cells were stained with fluorescent mAbs for relevant B cell molecules, and analyzed flow cytometrically prior to selecting the target sort population. Following geometric gating and exclusion of dead and CD3/CD14/CD16/CD235a+ cells, CD19+, surface IgD- colostrum B lymphocytes were selected as shown in this figure and sorted into 96 well plates. The colostrum CD19+ B cells were primarily negative for surface IgD expression and CD38−/+, consistent with memory B cells. CD5 expression of colostrum-derived B cells was largely absent.

Analysis of the flow cytometry data obtained during the sorting of colostrum B cells showed that they were primarily surface IgD-, consistent with a memory phenotype (Fig. 1). From 1876 single-sorted B cells, immunoglobulin gene RNA amplification resulted in 11 functional immunoglobulin gene pairs, of which seven were IgG and four were IgM. Two of the amplified and recombinantly-produced colostrum recombinant mAbs (18%) bound to gp120_Con.C_ in a screening standardized custom binding antibody multiplex assay [37] indicating a relatively high frequency of Env-specific B cells in this subject [38]. Both antibodies were IgG1 isotype (Table S1), though they were not clonally-related. The CH07 heavy chain had a mutation frequency of 2.2%; the CH07 light chain (lambda) had a mutation frequency of 2.0%. The heavy and light chain CDR3s of CH07 were hydrophilic (GRAVY values of -0.75 and -1.3, respectively), and the heavy chain was acidic (net charge -5) while the light chain was neutral (net charge 0). In contrast, CH08 was more mutated with a mutation frequency of 5.6% in the heavy chain and 1.2% in the light chain (kappa). The heavy and light chain CDR3s of CH08 were also hydrophilic (GRAVY values of -0.21 and -0.73, respectively) and acidic (net charge -3 and -2, respectively) (Table S1). Many antibodies with broad neutralizing activity have mutation high frequencies and/or are hydrophobic [39], [40], however, CH07 and CH08 had neither of these characteristics.

### Breadth of HIV-1 Env-specific colostrum mAb binding and neutralization

We next assessed the ability of mAbs CH07 and CH08 to bind to HIV-1 Env gp120 and gp140 proteins. CH07 bound to gp120 proteins, but did not bind to gp140 proteins, even at antibody high concentration (50 µg/mL, estimated EC_50_>200 nM). CH07 demonstrated strong binding to group M and clade C consensus gp120 proteins, and also bound with lower relative affinity to gp120_A244.CRF01_AE_ and gp120_MN.B_ (Table 1). In contrast, mAb CH08 bound to a broader range of Envs, displaying strong binding to consensus clade B and C gp140 Env proteins and weak binding to consensus clade CRF01_AE and G gp140 proteins, clade A and G primary isolates, and clade C and group M consensus gp120 proteins (Table 1). Neither antibody bound to gp41_MN.B_ or group A gp140.

**Table 1:** Relative binding affinity of mAbs CH07 and CH08 to Env proteins.

	mAb EC_50_ (nM)	
	CH07	CH08
**gp120s**		
Con.C	3.68	weak^1^
Con.6	1.01	weak
A244.CRF01_AE	98	–2
MN.B	weak	–
**gp140s** 3		
Con.B	–	13.5
Con.C	–	11.9
Con.G	–	weak
Con.CRF01_AE	–	weak
Con.6	–	65
00MSA.A	–	weak
DRCBL.G	–	130

1 weak = binding observed, but saturation not reached at a mAb concentration of 50 µg/mL (estimated EC_50_>200 nM).

2 – = no binding observed.

3 No binding was detected for either mAb to gp140_Con.A_, gp140_VRC.A_, gp140_VRC.B_, or gp41_MN.B_.

**Figure 2:**
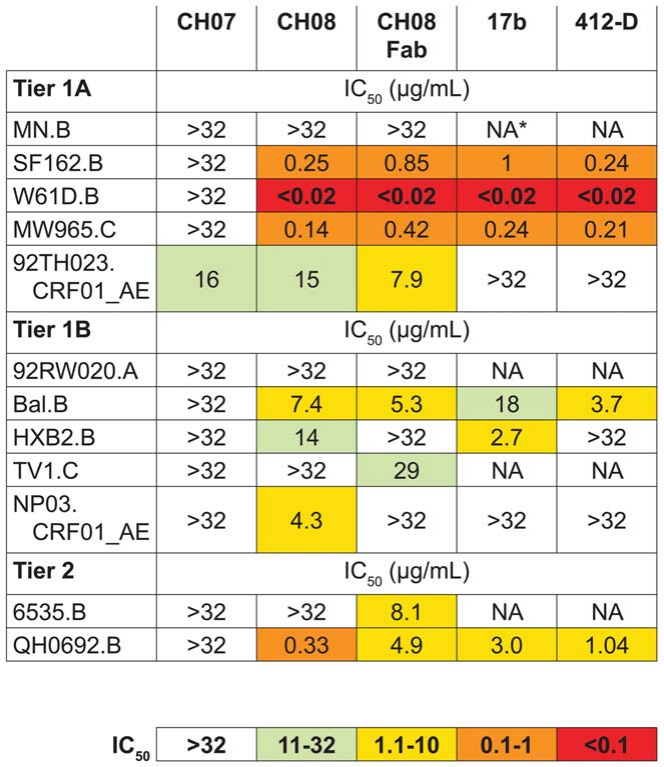
HIV-1 pseudovirus neutralization by mAbs CH07 and CH08. A panel of tier 1 and tier 2 HIV-1 pseudoviruses were tested against mAbs CH07, CH08, 17b, and 412-D in TZM-bl cells. CH07 weakly neutralized a single virus (92TH023.CRF01_AE). In contrast, CH08 neutralized multiple tier 1 and tier 2 viruses in a pattern similar to that of CD4i mAbs 17b and 412-D. A fragment (Fab) of CH08 neutralized with a potency similar to that of the intact mAb, suggesting that the binding site for mAb CH08 was not inaccessible for most of the isolates tested. An additional panel of 13 tier 2 viruses was not neutralized by any mAb tested.

Next, we studied the ability of the colostrum mAbs to neutralize HIV-1 in the TZM-bl Env pseudovirus assay. CH07 weakly-neutralized a single tier 1A clade CRF01_AE virus, 92TH023 (Fig. 2). In contrast, CH08 had broader neutralizing activity, potently neutralizing tier 1A viruses of clades B and C and less potently neutralizing tier 1A and 1B isolates of clade CRF01_AE (Fig. 2). CH08 also had activity against tier 1B and tier 2 clade B isolates that are more resistant to neutralization. This neutralization pattern is similar to previously-described CD4i gp120-specific antibodies, 17b and 412-D [24], [29] (Fig. 2). However, neither 17b nor 412-D neutralized clade CRF01_AE strains, demonstrating a greater breadth of activity for CH08. The CD4i mAb 17b has increased neutralization potency as a monovalent Fab fragment, likely due to the binding site being poorly accessible to the intact mAb [41]. This was untrue of CH08, as its Fab neutralized most of the same viruses as the intact CH08 with similar potency (Fig. 2).

### Additional anti-HIV-1 activity of mAbs CH07 and CH08

**Figure 3:**
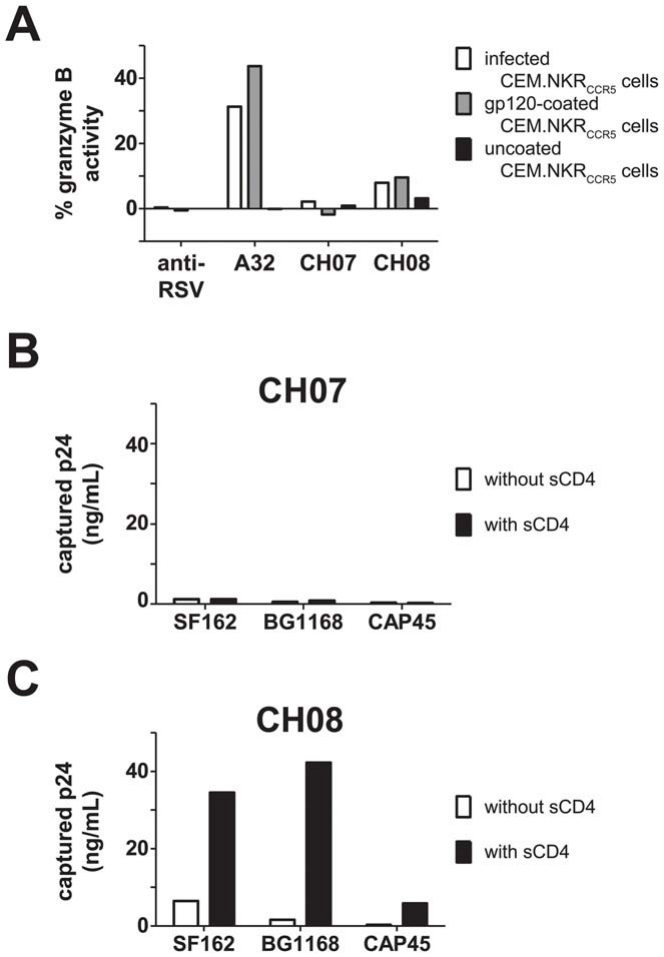
ADCC and virus capture by mAbs CH07 and CH08. A. CH07 and CH08 were tested for ADCC activity compared with anti-RSV mAb (Palivizumab) and A32. CH07 did not mediate ADCC, while both A32 and CH08 stimulated granzyme B activity NK effector cells when reacted with infected and gp120-coated targets. Each bar represents the percent of target cells containing granzyme B. B. CH07 did not capture pseudoviruses SF162.B, BG1168.B, and CAP45.C. Data shown as p24 released from captured virus. C. CH08 captured all three pseudoviruses in the presence of sCD4 but not when sCD4 was absent. Data shown as p24 released from captured virus.

We assessed the ability of CH07 and CH08 to mediate nonneutralizing anti-HIV functions antibody dependent cellular cytotoxicity (ADCC) and virus capture. CH07 did not mediate detectable ADCC, while CH08 mediated low level ADCC against both gp120 Env-coated and HIV-1-infected target cells (Fig. 3A) that was less potent than that of mAb A32, an antibody with common ADCC specificity in HIV-1 infected subjects (Fig. 3A)[42]. CH07 also had little virus capture activity (Fig. 3B). In contrast, CH08 captured clade B and C Env pseudoviruses, but was only able to do so in the presence of sCD4 (Fig. 3C), indicating that CH08 likely binds to a CD4i epitope.

### MAb CH07 binds to linear epitopes in the gp120 C5 domain and the gp41 fusion domain

**Figure 4:**
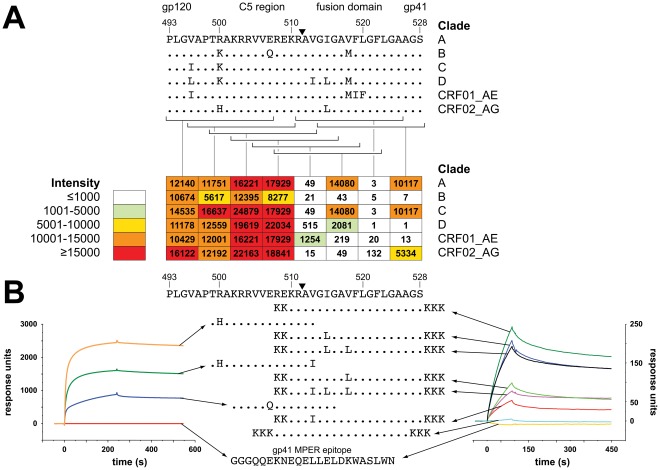
MAb CH07 binding to the HIV-1 Env gp120 C5 region and gp41 fusion domain is sequence specific. A. Peptide array binding of mAb CH07 is shown. Overlapping C5 and fusion domain peptide sequences are indicated by brackets, the strength of the peptide binding is indicated in the table below. The C5 and fusion domain sequence is labeled by HXB2 numbering and the gp41 cleavage site is indicated by a black triangle. CH07 bound strongly to peptides in the gp120 C5 region as well as to peptides in the gp41 fusion domain. B. Binding to variant fusion domain and C5 region peptides was measured by SPR. Binding of CH07 to peptides in the C5 region appeared to be most sensitive to a Glu-to-Gln (E to Q) change at position 507. In the fusion domain, hydrophobic amino acid changes at positions 513, 515, and 518 changed binding, suggesting that each substitution altered the conformation of the region in a manner that reduced the ability of CH07 to bind. A consensus sequence peptide containing additional amino acids in the C5 region did not bind, indicating that overlap with C5 sequence was not responsible for the reactivity seen. For both SPR experiments, a gp41 membrane proximal external region peptide was used as a negative control.

The CH07 Env binding pattern suggested that its binding site was more accessible in gp120 than gp140 constructs. Using a linear peptide array, CH07 appeared to bind two distinct regions of HIV-1 Env flanking the gp41 cleavage site. The strongest CH07 binding was to peptides comprising the C5 region of gp120 (Fig. 4A). Conservative amino acid substitutions in this region had limited effects on binding, with the exception of a Glu-to-Gln change near the Env cleavage site that reduced, but did not eliminate binding (Fig. 4A). CH07 bound also to two non-overlapping fusion domain peptides at the N-terminus of gp41 and had weak binding to a third peptide centered on the Env cleavage site (Fig. 4A).

To further investigate the apparent bivalent Env binding of CH07, we constructed C5 and fusion domain peptides with amino acid substitutions that maintained net charge and hydrophilicity to assess CH07 binding by SPR (Fig. 4B). Three peptides spanning the C5 domain were generated (charge: +4.1 to +5.0 at pH 7.0; hydrophilicity: 1.0–1.4) (Fig. 4B). A Val-to-Ile substitution at the C-terminus of this region reduced binding modestly, while a Glu-to-Gln substitution decreased the binding more substantially. A series of seven peptides overlapping the gp41 fusion domain (charge: +7 to +8 at pH 7.0; hydrophilicity: 0.2–0.6) revealed that binding was slightly decreased by an Ile-to-Leu change near the Env cleavage site (Fig. 4B). A more dramatic decrease in binding was observed with combinations of Val-to-Ile/Leu changes, suggesting that sequence changes may alter the binding site through conformational changes. Interestingly, no binding was detected to a peptide that included residues that bridged the region between the C5 and fusion domain binding sites additional residues in C5, confirming that mere overlap was not responsible for the bivalent binding pattern (Fig. 4B).

### CH08 recognizes a CD4i epitope

**Figure 5:**
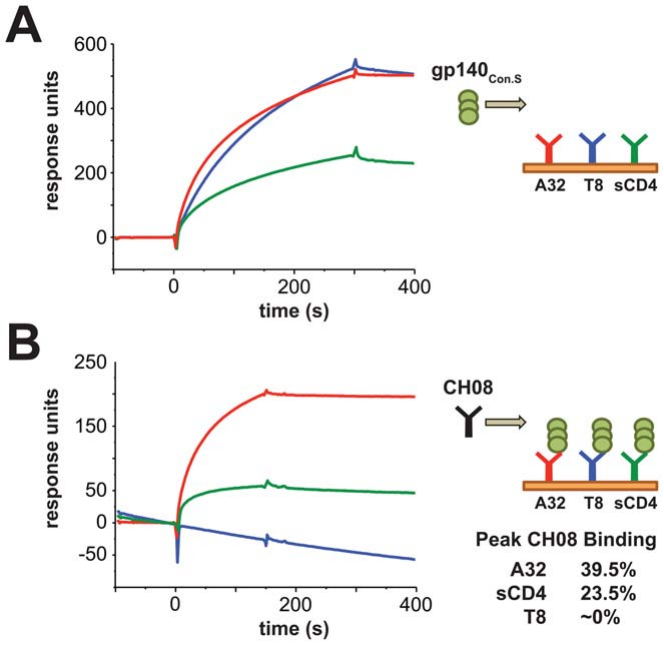
MAb CH08 is a CD4i HIV Env-specific antibody. CH08 mAb binding to gp140_Con.S_ after capture by mAb T8 (HIV gp120-specific mAb), soluble CD4 (sCD4), or mAb A32 (CD4 binding site mAb) was measured by SPR. A. gp140_Con.S_ bound to anchored T8, sCD4 and A32, data are shown in response units (RU). B. CH08 was flowed over the captured Env showing that it bound to Env captured by A32 and sCD4, but not Env captured by mAb T8. Peak CH08 binding as a fraction of bound Env is shown.

CH08 did not bind to linear epitopes of gp160 (not shown), so the ability of CH08 to bind conformational Env proteins was measured by SPR. We anchored Env-binding molecules (sCD4, and mAbs T8 and A32) to a chip and flowed Env gp140_Con.S_ over them (Fig. 5A). Env bound to mAb T8 retains its native conformation, whereas Env bound to mAb A32 undergoes a conformational change that exposes the binding site for the CD4i mAb 17b [24]. MAbs T8 and A32, and sCD4 all captured gp140 (Fig. 5A). In a second step, mAb CH08 was flowed over the captured gp140_Con.S_ (Fig. 5B). CH08 did not bind to gp140_Con.S_ captured by mAb T8, displayed in a neutral conformation. In contrast, CH08 bound strongly to gp140_Con.S_ captured by sCD4 and mAb A32, consistent with CH08 binding to a post-liganded, CD4i epitope of gp120 (Fig. 5B). Moreover, CD4i mAbs specific for the CCR5 binding site, 17b and 412-D [29], [43], [44], inhibited the binding of CH08 to the CD4-gp120 complex in blocking ELISA assays (Fig. S1).

### CH08 is a sulfated antibody

Many CD4i antibodies, including 412-D, have been shown to contain sulfated tyrosine residues that mimic the site on CCR5 that interacts with gp120, allowing those mAbs to bind Env in a manner similar to CCR5 [40], [45]. A single sulfation site was predicted within the CDRH3 of mAb CH08 (Table S1). Moreover, western blotting of a reduced gel of mAbs CH07, CH08, and 412-D stained with an anti sulfo-tyrosine mAb demonstrated bands for both 412-D and CH08, but not CH07, indicating the CH08 has at least one sulfated tyrosine residue (Fig. S2).

### CH07, but not CH08, has strong polyreactivity

**Figure 6:**
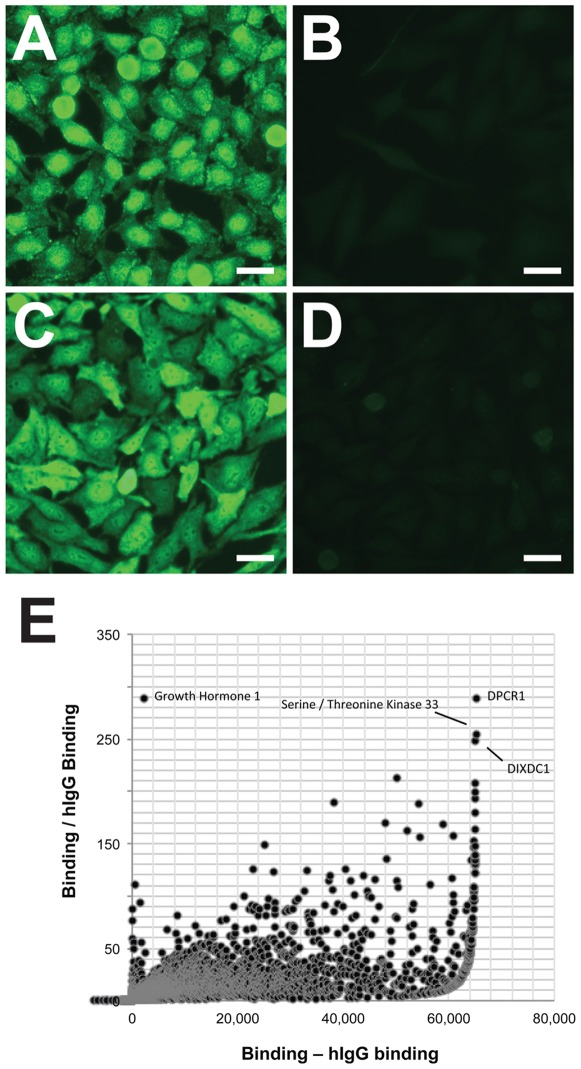
Autoreactivity of mAb CH07. A–D. Indirect immunofluorescence staining of HEp-2 cells was strongly positive for mAbs CH07 (A, diffuse nuclear and clumped peripheral staining pattern) and 2F5 (C, diffuse cytoplasmic and nuclear staining pattern); mAbs CH08 (B) and 17b (D) were negative. All images taken for 10 s except CH07 taken for 7 s. Size bars are 25 µm. E. Reactivity of mAb CH07 to 9,000 human proteins in a microarray was assessed, each spot represents binding to a single protein. Pooled human IgG (hIgG) was used to measure background binding. The X-axis represents antibody binding minus background. The Y-axis represents antibody binding divided by the background binding. Positive signals for specific proteins are indicated by labels.

The polyreactivity of many neutralizing anti-HIV-1 Env antibodies is well-documented [34], [46]. In fact, the rarity of HIV-1-broadly neutralizing antibodies is thought to be due in part to the clonal deletion of B cells that produce antibodies that bind to multiple antigens, including self-antigens, and are also able to neutralize HIV-1 through heteroligation. Pregnancy is a state in which cellular immunity is suppressed in order to avoid rejection of the non-self fetus, increasing the possibility of autoreactive B cells escaping deletion during this time. Thus, we assessed the polyreative status of CH07 and CH08. CH08 showed no reactivity against any standard clinical autoantigens, whereas CH07 reacted with SCL70, double stranded DNA, and centromere B (data not shown). In a HEp-2 cell line, CH08 also showed no binding (Fig. 6B), whereas CH07 displayed strong binding with diffuse nuclear staining and clumped peripheral staining pattern (Fig. 6A). This reactivity did not appear to be associated with lipid binding, as neither antibody bound to cardiolipin or phosphotidylserine preparations (data not shown). Finally, we observed that CH07 reacted with over 6000 human proteins on a protoarray autoantigen chip (Fig. 6E). Thus, mAb CH07 is highly-polyreactive, perhaps explaining its unusual Env-specific binding to the disparate C5 and fusion domains.

## Discussion

We identified two anti-HIV-1 Env mAbs of mucosal origin through B cell isolation from colostrum which displayed HIV-1-neutralizing activity. These mAbs represent two of the first HIV-1 Env-specific mAbs to be isolated from a mucosal compartment, and the first to be isolated from colostrum. As most HIV-1 transmission occurs via mucosal barriers, the study of effective mucosal B cell responses is critical to the design of HIV-1 vaccine candidates that will elicit protective antibodies from mucosal B cell populations. This study represents an initial step in determining the breadth and function of HIV-1 Env-specific antibodies that are inducible in mucosal compartments.

MAb CH07 is a highly autoreactive antibody that binds to linear epitopes within the gp120 C5 region and gp41 fusion domain and displays weak neutralization against a clade AE virus. The C5 domain is a hydrophilic region within gp120 that roughly 80% of HIV-1-infected individuals make antibodies against [47], [48]. In contrast, the fusion domain is a hydrophobic region of gp41 that is essential for viral fusion [49]. Remarkably, no anti-HIV-1 Env mAbs reported to date are specific for this region. It may not be surprising, however, that this antibody was isolated from a woman who was recently pregnant. The cellular immune system of a pregnant woman is suppressed in order to prevent immune-mediated rejection of the fetus. The precise mechanisms of this suppression are poorly understood, however, in the combined state of pregnancy and HIV-1 infection, autoreactive B cells may be subject to less stringent tolerance control and may not be deleted as efficiently. This setting may make the appearance of polyreactive anti-HIV-1 antibodies more likely, but it is unknown if such antibodies are common among HIV-1-infected pregnant women. Due to the weak neutralization and strong autoreactivity of mAb CH07, CH07-like mucosal antibody responses are not likely a suitable vaccine target in the general population. However, it is also possible that the use of HIV-1 vaccine candidates in both infected and uninfected pregnant women may result in an altered, and possibly more robust, HIV-1-neutralizing antibody responses compared to nonpregnant individuals, but at this time such surmises remain speculative.

The identified colostrum mAb CH08 is a CD4i mAb with moderate-breadth HIV-1-neutralizing activity, representing a potential target for transmission-blocking mucosal responses. CH08 shares many traits with the subclass of CD4i antibodies including 1–69 V_H_ gene usage, long acidic CDR loop, and a sulfated tyrosine within the CDR region [40]. However, neutralization studies show that CH08 has slightly broader and more potent neutralization capacity than other CD4i mAbs, indicating a potentially more accessible gp120 binding site. Structural characterization of the CH08 binding site may be important for guiding efforts to elicit these types of antibodies in uninfected individuals. Importantly, CH08 was not polyreactive, and thus vaccine induction of CH08-like mucosal antibodies may be feasible.

Recent nonhuman primate and human studies of HIV-1-specific immune responses in breast milk have indicated that virus-specific IgG primarily mediates the neutralizing and non-neutralizing responses in milk, despite the total antibody pool in milk being mainly comprised of IgA [13], [14]. It is therefore not surprising that these HIV-1 Env-specific colostrum mAbs, CH07 and CH08, are IgG isotype, and this is consistent with previous work demonstrating that IgG-secreting B cells in milk predominate over IgA-secreting B cells [9]. Further characterization of locally-produced anti-HIV-1 functional IgG responses in milk may illuminate why the majority of nursing infants of HIV-1-infected infants are protected against HIV-1 acquisition, despite chronic, daily mucosal HIV-1 exposure. In fact, mucosal IgG responses, rather than mucosal IgA responses, may be a more appropriate target of maternal vaccines aimed at interrupting postnatal HIV transmission.

## Supporting Information

Figure S1 **MAb CH08 binding to HIV envelope is blocked by V3-binding CD4i antibodies 17b and 412D.** Blocking of biotinylated mAb CH08 binding to ConS gp140 was assessed against CH08 (A), 412-D (B), 17b (C), and 21c (D). X-axis shows the concentration of the blocking antibody added. The Y-axis shows the percent of CH08 that bound in the presence of the blocking antibody compared to the binding of CH08 in the absence of blocking antibody. Error bars are standard error of the mean of results from quadruplicate (CH08, 412-D, and 17b) or duplicate (21c) assays.
(PDF)

Figure S2 **MAb CH08 contains a sulfation site within the CDR3 region.** Western blot of mAbs 412-D (positive control), CH08, and CH07 blotted with an anti-sulfotyrosine mAb. Bands at ∼50 kDa in the reduced gel represent at least one positive sulfated tyrosine in the heavy chains of CH08 and 412-D, but not CH07.
(PDF)

Table S1 **MAbs CH07 and CH08 heavy and light chain and CDR3 characteristics.**
(DOCX)
