# Supplementary figures and images for: Isolation of HIV-1-Neutralizing Mucosal Monoclonal Antibodies from Human Colostrum
Source: PLoS One. 2012 May 18;7(5):e37648. doi: 10.1371/journal.pone.0037648 (PMC3356285; doi:10.1371/journal.pone.0037648)

Supplemental Fig 1

**A**

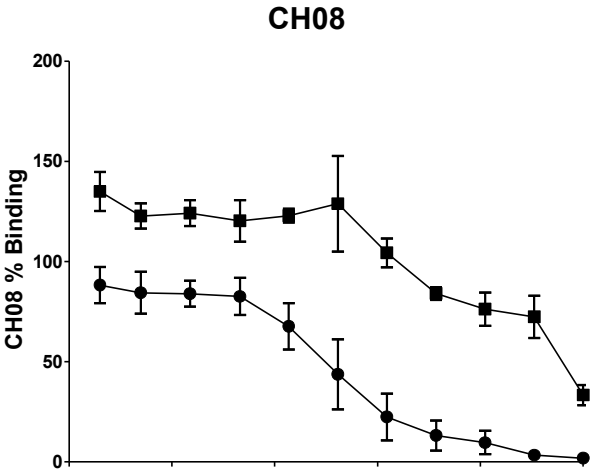

**B**

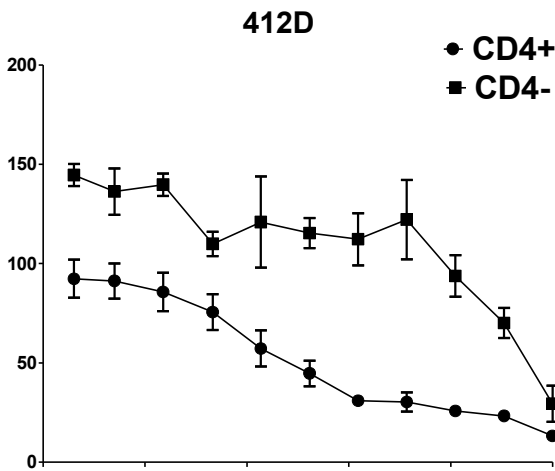

**C**

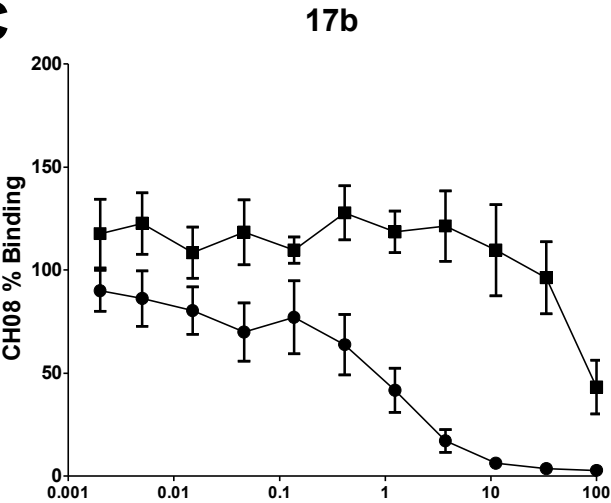

**D**

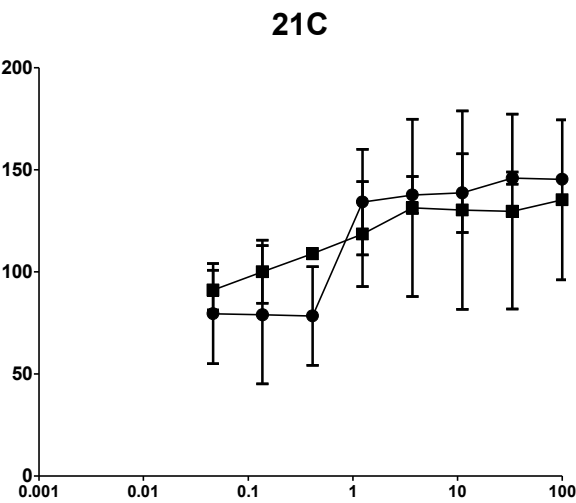

Concentration  $\mu\text{g/mL}$

Supplement: Figure S1 — MAb CH08 binding to HIV envelope is blocked by V3-binding CD4i antibodies 17b and 412D. Blocking of biotinylated mAb CH08 binding to ConS gp140 was assessed against CH08 (A), 412-D (B), 17b (C), and 21c (D). X-axis shows the concentration of the blocking antibody added. The Y-axis shows the percent of CH08 that bound in the presence of the blocking antibody compared to the binding of CH08 in the absence of blocking antibody. Error bars are standard error of the mean of results from quadruplicate (CH08, 412-D, and 17b) or duplicate (21c) assays. (PDF) [file pone.0037648.s001.pdf]

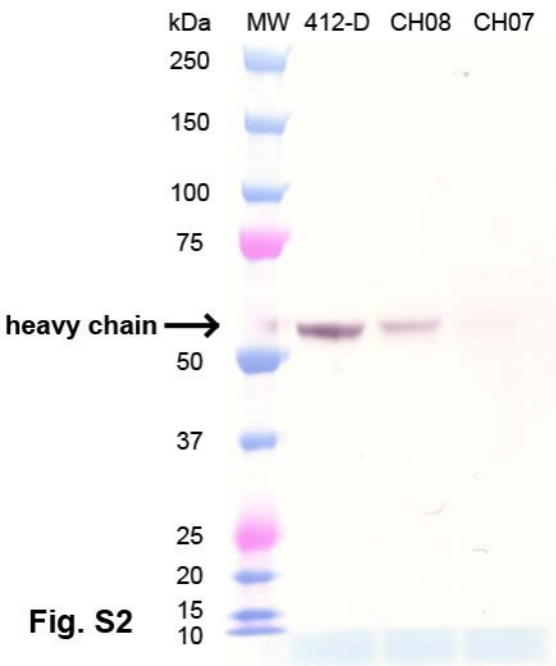

Supplement: Figure S2 — MAb CH08 contains a sulfation site within the CDR3 region. Western blot of mAbs 412-D (positive control), CH08, and CH07 blotted with an anti-sulfotyrosine mAb. Bands at ∼50 kDa in the reduced gel represent at least one positive sulfated tyrosine in the heavy chains of CH08 and 412-D, but not CH07. (PDF) [file pone.0037648.s002.pdf]
