# Supplementary material for: Isolation of HIV-1-Neutralizing Mucosal Monoclonal Antibodies from Human Colostrum
Source: PLoS One. 2012 May 18;7(5):e37648. doi: 10.1371/journal.pone.0037648 (PMC3356285; doi:10.1371/journal.pone.0037648)
Supplement: Table S1 — MAbs CH07 and CH08 heavy and light chain and CDR3 characteristics. (DOCX) [file pone.0037648.s003.docx]

Supplemental Table 1: **MAbs CH07 and CH08 heavy and light chain and CDR3 characteristics.**

| Name | Type/ Gene Usage | Mutation Frequency | CDR3 Sequence | CDR3 length | GRAVY | Net CDR3 Charge |
| --- | --- | --- | --- | --- | --- | --- |
| **CH07** | IgG1 |  |  |  |  |  |
| Heavy Chain | 3-15*01 | 2.20% | TTEPDSGTYYNLSVFDY | 17 | -0.753 | -5 |
| Light Chain | Lambda | 1.90% | YSTDSSDNPL | 10 | -1.27 | 0 |
|  | 3-10*01 |  |  |  |  |  |
| **CH08** | IgG1 |  |  |  |  |  |
| Heavy Chain | 1-69*01 | 5.60% | ATGSDFDY^1^GGDSGIGVDFDF | 20 | -0.21 | -3 |
| Light Chain | Kappa | 1.20% | QQLNSYPIT | 9 | -0.733 | -2 |
|  | 1-9*01 |  |  |  |  |  |

^1^ Indicates predicted sulfation site.
